# Impact on physical function of the +AGIL Barcelona program in community-dwelling older adults with cognitive impairment: an interventional cohort study

**DOI:** 10.1186/s12877-023-04292-4

**Published:** 2023-11-13

**Authors:** Cristina Arnal, L Monica Pérez, Luís Soto, Álvaro Casas Herrero, Joan Ars, Sonia Baró, Francisco Díaz, Araceli Abilla, M Belén Enfedaque, Matteo Cesari, Marco Inzitari

**Affiliations:** 1 RE-FiT Barcelona Research group. Vall d’Hebron Institute of Research (VHIR) and Parc Sanitari Pere Virgili, Barcelona, Spain; 2 Department of Medicine, Autonomous University of Barcelona, Barcelona, Spain; 3 Geriatric Department, Hospital Universitario de Navarra (HUN), Pamplona, Spain; 4 Navarrabiomed, Hospital Universitario de Navarra(HUN), Universidad Pública de Navarra(UPNA), IdiSNA, Pamplona, Spain; 5 CIBER of Frailty and Healthy Aging (CIBERFES), Instituto de Salud Carlos III, Madrid, Spain; 6 Primary Healthcare Center Larrard, Primary Care Pere Virgili and PiC research group of the IMIM, Barcelona, Spain; 7 Primary Healthcare Center Bordeta-Magòria, Catalan Health Institute, Barcelona, Spain; 8 Catalan Health Institute, Barcelona, Spain; 9 Geriatric Unit, IRCCS Insittuti Clinici Scientifici Maugeri, University of Milan, Milan, Italy; 10 Faculty of Health Sciences, Open University of Catalonia (UOC), Barcelona, Spain

**Keywords:** Community-dwelling, Aged, Cognition disorders, Physical functional performance, Multicomponent exercise program.

## Abstract

**Background:**

Older adults with cognitive impairment (CI) have higher multimorbidity and frailty prevalence, lower functional status and an increased likelihood to develop dementia, non-cognitive deficits, and adverse health-related events. +AGIL, a real-world program for frail older adults in a primary care area of Barcelona, is a pragmatic, multi-component and integrated intervention implemented since 2016. It includes physical activity, nutrition, sleep hygiene, revision and adequacy of pharmacological treatment, detection of undesired loneliness and screening for CI; to improve physical function in community-dwelling older adults. We aimed to assess the + AGIL longitudinal impact on physical function among community-dwelling frail older persons with CI.

**Methods:**

An interventional cohort study included data from all the + AGIL consecutive participants from July 2016 until March 2020. Based on the comprehensive geriatric assessment, participants were offered a tailored multi-component community intervention, including a 10-week physical activity program led by an expert physical therapist. Physical performance was measured at baseline, three and six months follow-up. The pre-post impact on physical function was assessed by paired sample t-test for repeated samples. Linear mixed models were applied to analyze the + AGIL longitudinal impact. P-values < 0.05 were considered statistically significant.

**Results:**

194 participants were included (82 with CI, based on previous diagnosis or the Mini-COG screening tool), 68% women, mean age 81.6 (SD = 5.8) yo. Participants were mostly independent in Activities of Daily Living (mean Barthel = 92.4, SD = 11.1). The physical activity program showed high adherence (87.6% attended ≥ 75% sessions). At three months, there was a clinically and statistically significant improvement in the Short Physical Performance Battery (SPPB) and its subcomponents in the whole sample and after stratification for CI [CI group improvements: SPPB = 1.1 (SD = 1.8) points, gait speed (GS) = 0.05 (SD = 0.13) m/s, Chair stand test (CST)=-2.6 (SD = 11.4) s. Non-CI group improvements: SPPB = 1.6 (SD = 1.8) points, GS = 0.08 (SD = 0.13) m/s, CST=-6.4 (SD = 12.1) seg]. SPPB and gait speed remained stable at six months in the study sample and subgroups. CI had no significant impact on SPPB or GS improvements.

**Conclusion:**

Our results suggest that older adults with CI can benefit from a multidisciplinary integrated and comprehensive geriatric intervention to improve physical function, a component of frailty.

## Background

Frailty is a syndrome characterized by increased vulnerability to stressors, leading to a higher risk of adverse health outcomes (e.g. disability, falls, fractures, institutionalization, and death) [1]. Older adults with cognitive impairment (CI) often present multimorbidity, frailty [2] and poor functional status. Not surprisingly, they are exposed to an increased risk of developing dementia and adverse health-related outcomes [3, 4, 5, 6, 7].

Different studies have described cross-sectional and longitudinal associations between frailty and CI [8, 9, 10]. The early identification and management of frailty and cognitive deficits are indeed critical to prevent the risk of further physical and/or cognitive declines. Unfortunately, older persons, especially those presenting CI, are often excluded from clinical trials and gold-standard clinical interventions [11].

Several non-pharmacological interventions, including lifestyle changes, have been proposed to reduce the impact of frailty and CI in older adults [12]. Physical exercise (PE) and physical activity (PA) have demonstrated positive effects on health-related outcomes [13, 14, 15] (e.g., improvement in physical function, mood, anxiety, sleep quality, falls, cardiovascular diseases) in the general population, including older adults. Unfortunately, since older adults with CI may perform worse in physical function tests, most previous studies testing PE and PA interventions have frequently excluded them [16]. Consequently, this population is largely underrepresented, and the available evidence shows heterogeneous results [17, 18]. Recent evidence suggests that people with moderate-severe CI and dementia are still likely to positively respond to and benefit from exercise [18, 19], especially multicomponent exercise training [20].

Together with PA, pharmacolocgic adequacy, malnutrition and sleep disturbances are major modifiable risk factors for CI and frailty onset and accentuation [21]. Previous studies have shown that lifestyle modifications (e.g., adherence to the Mediterranean diet [22, 23]) can reduce the risk of future cognitive impairment, dementia and frailty [24, 25, 26]. Additionally, sleep disturbances may predict the risk of incident dementia and frailty. An individualized medication review in frail older patients can adverse drug effects, such as polypharmacy, therapeutical complexity, and anticholinergic or sedative burden [27]. However, research is needed to evaluate whether targeting only sleep disturbance may be an opportunity for new dementia treatment or frailty prevention.

The + AGIL Barcelona program is a pragmatic, sustainable, ongoing multicomponent intervention [28]. Its multifactorial intervention addresses physical exercise, nutrition, sleep quality, optimization of pharmacological treatment, health education, and cognitive evaluation. The program pursues the translation of evidence from RCTs to implementation in the community. Its main aim is to improve physical function in older adults with initial frailty through an integrated care approach between primary care, geriatrics, and community resources. It is also designed to empower participants, fostering their engagement in community activities. This program has shown to be effective in older adults with different degrees of frailty [29].

This article aims to assess the longitudinal impact on physical function among community-dwelling older people with confirmed or possible CI. In particular, we are interested in verifying whether the effects of the + AGIL Barcelona program may differ according to the presence/absence of CI.

## Methods

### Study population

Data are from the + AGIL Barcelona program [28]. Briefly, +AGIL is an ongoing co-designed program implemented in Barcelona since July 2016. It aims to prevent, detect and revert frailty in community-dwelling older adults. +AGIL Barcelona’s main characteristics are: (a) it is based on the integration and coordination between primary care, geriatrics teams and community resources; (b) it offers an individualized, adaptable, flexible and person-centred plan based on the Comprehensive Geriatric Assessment (CGA); (c) it counts with the end user’s active participation from the beginning, through co-design strategies; (d) it promotes sustainability overtime, through participants’ empowerment and implication of community resources and digital components. The program was effective to improve physical function at three months (i.e. Short Physical Performance and gait speed), also in participants with different levels of frailty at the baseline [29].

The current study included data from all the consecutive participants from July 2016 until March 2020 who performed at least one follow-up visit (3 or 6 months).

### Intervention

After identifying potential intervention beneficiaries through the Gerontopôle Frailty Screening Tool (GFST), the Primary Care teams refer the person to the Geriatric team (i.e., a geriatrician and physical therapist) working in the primary care center. The team then offers a tailored multi-component intervention based on the results of the CGA. The plan, extensively reported elsewhere [28], includes:

- After a first individual assessment, a 10 weeks boost of multicomponent exercise (MEP) is designed, adapted and recommended by an expert physical therapist. The MEP includes strength, resistance, balance and coordination exercises to be performed at home by themselves and an onsite supervised program run by the therapist once a week during 1 h. When possible, the home MEP is performed through a digital component to empower participants in the regular conduction of PE and increase PA levels through the validated ViviFrail© platform. ViviFrail© has been designed to prevent frailty and falls in older adults through a personalized multicomponent exercise program [30]. This program has shown relevant benefits in terms of functional and cognitive improvements in older hospitalized adults [31] and very recently in frail community-dwelling older adults with cognitive decline or mild dementia [19]. The MEP onsite sessions include 10 min of warm-up, 15 min of strength training, 10 min of balance training, 10 min of resistance training, 5 min of flexibility training and 5 min of stretching exercises, modulated according to the participants’ capacity.
- An intervention aimed at promoting adherence to a Mediterranean diet, following Prevention with Mediterranean diet (PREDIMED) intervention paradigm [32].
- Other non-pharmacological interventions directed to promote healthy habits, reinforcing the ongoing activities in the primary care center(e.g. improving sleep hygiene, smoke or alcohol control, PE community groups, etc.);
- Screening of cognitive decline or non-desired loneliness plus coaching based on motivational interviewing;
- Pharmacological optimization. A comprehensive review of the medication is done with a clinical pharmacist’s remote support, focusing on identifying and withdrawing inappropriate medication based on patient goals, and validated tools (i.e. STOPP/START [33], Beers Criteria [33, 34]) mainly focus on stopping psychotropic drugs. Modifications or deprescribing processes are made in agreement with the family physician and the participant.

### Cognitive assessment

The Mini-Cog© (Washington, DC, USA) was used in all the participants for CI screening. It is a 2-component instrument (i.e. a 3-item recall test and clock-drawing test). The Mini-Cog© evaluates cognitive function and memory, language comprehension, visual-motor skills, and executive function [35]. It has been validated to use in primary care settings [36]. Results (range 0–5) scoring under three indicates positive screening for cognitive impairment.

### Covariates

We collected sociodemographic data, clinical characteristics (including the Charlson Comorbidity Index [37]), and current treatment. Data retrieved from the CGA include the basic activities for daily living (ADLs) and instrumental activities of daily living (IADLs), measured with the Barthel Index (BI, range from 0 [completely dependent] to 100 [completely independent]) [38] and the Lawton-Brody Instrumental Activities of Daily Living Scale (range from 0 [completely dependent] to 8 [completely independent]) [39], respectively.

### Outcomes

Physical function was assessed using the Short Physical Performance Battery (SPPB) [40]. The SPPB includes three timed tests: (a) the 4-meter gait speed (GS), (b) the balance assessment in three different positions (straight, semi tandem and tandem), and (c) the chair stand test. Each test is scored from 0 to 4 points, with a total score ranging from 0 (worst physical function) to 12 points (best physical function). Previous studies have consistently described SPPB scores under 10 as a strong predictor of disability and the main proxy for frailty in non-disabled older adults [41].

### Statistical analysis

Baseline characteristics of the sample are presented as mean values and Standard Deviation (SD) for continuous variables, median values and interquartile range (IQR) for continuous and ordinal variables and frequency and percentages for categorical variables. The Student’s t-test or the Mann–Whitney U-test and Chi-square test were used as appropriate to analyze the possible differences among participants (a) included in the present analysis and those excluded for the absence of follow-up visit, and (b) those with and without CI (i.e. Mini-Cog© total score < 3 points or clinical diagnosis of mild cognitive impairment or dementia).

The pre-post impact on physical function at three and six months was assessed using the paired sample t-test for repeated samples, Wilcoxon signed-rank for continuous variables, and the McNemar’s test for categorical variables. According to previous studies [29], we imputed the value of 61 s for those participants unable to perform the chair stand test (n = 26). The presence of balance impairment was considered positive when the participant could not achieve the 4 points in the balance sub-item from the SPPB test.

Finally, the longitudinal association between the presence of CI and the impact on physical function (i.e. change in SPPB total score and gait speed and chair stand test subitems) was assessed using linear mixed models. The interaction between time and the primary exposure was included as a fixed effect. The resulting β coefficients can be interpreted as the effect of having any degree of cognitive impairment on the average monthly change of physical function indicators after completing the + AGIL Barcelona intervention. Random effects were defined for the intercept and slope, unstructured covariance was assumed, and restricted maximum likelihood estimation was applied. Models were adjusted for age, sex, education level and, previous functional capacity.

In all analyses, p-values < 0.05 were considered statistically significant. Analyses were performed using Stata version 14.

## Results

**Fig. 1:**
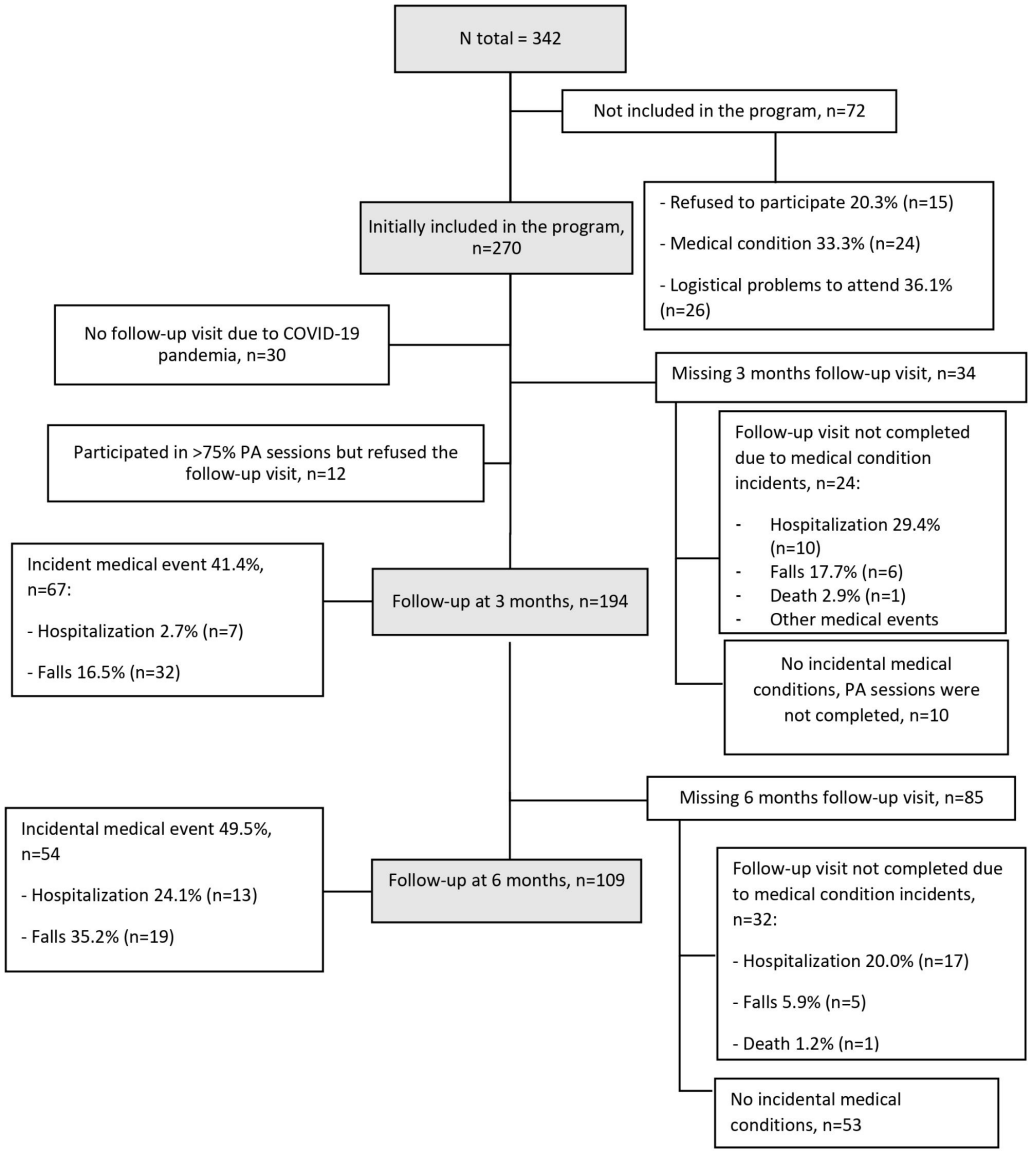
Population flowchart

Of the 342 people screened, 270 (78.9%) were invited and accepted to participate in the program. Reasons for non-participation of the 72 (21%) remaining candidates were diverse (15 refused to participate, 24 due to medical conditions, 26 due to logistical personal problems to attend, and 7 due to severe physical dependence). Among the participants, 76 did not attend the 3-month follow-up (30 due to COVID-19 lockdown restrictions, 23 due to medical events, 12 refused to undergo the follow-up visit after correctly completing ≥ 75% of physical activity sessions without any incident event, 10 did not complete the program and did not attend the follow-up visit despite the absence of medical complications, and one died) and were excluded, resulting in a sample of 194 participants, 82 in the CI group and 112 in the reference group. Baseline characteristics were comparable between the 194 patients included and the 148 excluded, Fig. 1.

Among all the participants, the mean age was 81.6 years (SD 5.8), 68% were women, and 39.4% lived alone *(*Table 1*)*. Most had primary (40.4%) or secondary (40.9%) education. Both groups presented relatively high comorbidity (median Charlson Index = 5.6 (SD 1.8)). In terms of physical function, most of the 194 participants were generally independent for ADL and IADL. Half of them reported at least one fall during the last year, and presented a low physical performance, according to SPPB and gait speed. A high proportion showed polypharmacy, with a mean of almost 8 drugs. Between the two groups of interest (CI and non-CI), there were only significant basal differences in terms of Barthel and Lawton index (the CI group showing more disability) and gait speed (slower for the CI group).

**Table 1:** Descriptive analysis of the population stratified by cognitive function

	Total (n = 194*)	Cognitive impairment (n = 82)	Non-cognitive impairment (n = 112)	P
**Age; mean (SD)**	81.6 (5.8)	82.3 (5.3)	81.0(6.1)	0.114
**Female; n (%)**	132 (68.0)	54 (65.9)	78 (69.6)	0.576
**Marital status; n (%)**:				0.347
Married Divorced Single Widow	85 (43.8) 5 (2.6) 20 (10.3) 84 (43.3)	41 (50.0) 1 (1.2) 9 (11.0) 31 (37.8)	44 (39.3) 4 (3.6) 11 (9.8) 53 (47.3)	
**Lives alone; n (%)**	76 (39.4)	28 (34.2)	48 (43.2)	0.201
**Educational level; n (%)**:				0.106
Analphabet Elemental Secondary University	10 (5.2) 78 (40.4) 79 (40.9) 26 (13.5)	8 (9.8) 32 (39.0) 32 (39.0) 10 (12.2)	2 (1.8) 46 (41.4) 47 (42.3) 16 (14.4)	
**Barthel index** ^**a**^; **mean (SD)**	92.4 (11.1)	90.4 (10.2)	93.9 (7.1)	**0.005**
**Lawton index; mean (SD)**	5.29 (2.5)	4.30 (2.62)	6.07 (2.10)	**0.000**
**Charlson index** ^**b**^; **mean (SD)**	5.6 (1.8)	5.8 (1.9)	5.4 (1.7)	0.136
**Number of drugs** ^**c**^; **mean (SD)**	7.7 (3.4)	7.9 (3.5)	7.7 (3.2)	0.376
**Falls in the last year; n (%)**	92 (47.4)	41 (50.0)	51 (45.5)	0.538
**SPPB** ^**d**^; **mean (SD)**	7.38 (2.36)	7.0 (2.12)	7.66 (2.61)	0.057
**Gait speed (m/s); mean (SD)**	0.70 (0.20)	0.67 (0.19)	0.73 (0.20)	**0.041**
**Chair Stand Test (s); median (IQR)**	23.95 (15.7)	23.05 (13.80)	24.60 (15.69)	0.500
**Balance impairment; n (%)**	101 (52.1)	44 (53.7)	57 (50.9)	0.703

*Sample with available 3-months follow-up

IQR: Interquartile Range. SD: Standar Desviation

^a^ Barthel index: range from 0-100. ^b^ Charlson index, range from 0–8. ^c^ Polypharmacy is defined as more than 5 drugs. ^d^ Short Physical Performance Battery, range from 0–12 points, < 10 points frailty indicator

Adherence to the MEP was high (87.6% attended ≥ 75% of sessions, mean [SD] = 8.9 [SD 2.1] out of 10 planned sessions). Participants reported similar rates regarding adherence to health and nutritional recommendation. After 3-months, an improvement in all the physical performance measures was reported in both groups (CI and non-CI) *(*Table 2*)*.

**Table 2:** Effect of the Multifactorial Intervention on physical performance at 3 and 6 months. Analysis stratified by presence of cognitive impairment (CI).

	3-months follow-up						6-months follow up					
	CI (n = 82)			Non CI (n = 112)			CI (n = 48)			Non CI (n = 61)		
	*Baseline*	*3 m f-up*	*P-value*	*Baseline*	*3 m f-up*	*P-value*	*Baseline*	*6 m f-up*	*P-value*	*Baseline*	*6 m f-up*	*P-value*
**SPPB; mean (SD)**	7.1 (2.1)	8.2 (2.5)	**< 0.001**	7.6 (2.5)	9.2 (2.3)	**< 0.001**	6.8 (1.9)	7.7 (2.6)	**0.005**	7.9 (2.5)	9.2 (2.9)	**< 0.001**
**Gait speed (m/s); mean (SD)**	0.67 (0.19)	0.72 (0.19)	**< 0.001**	0.73 (0.2)	0.81 (0.19)	**< 0.001**	0.65 (0.17)	0.69 (0.19)	**0.048**	0.76 (0.21)	0.81 (0.23)	**0.010**
**Chair Stand Test (s); mean (SD)**	22.6 (13.2)	20.0 (14.5)	**0.049**	25.1 (17.3)	18.7 (14.5)	**< 0.001**	24.4 (14.1)	24.1 (19.0)	0.860	25.8 (18.1)	19.1 (15.9)	**0.002**
**Balance impairment, n (%)**	43 (53.1)	33 (40.7)	**0.025**	57 (51.4)	37 (33.3)	**< 0.001**	24 (52.1)	26 (56.5)	0.617	25 (41.7)	18 (30.0)	0.090

SD: standard deviation; a. SPPB: Short Physical Performance Battery, range from 0 to 12. Paired sample t-test for repeated samples or Wilcoxon signed-rank were used for continuous variables as appropriate and Mc Nemar’s test for categorical variables. For the “balance impairment and falls categories, we report the n (%) of participants with the event

Overall, 109 participants attended the 6-month follow-up assessment, in whom the improvement of SPPB and gait speed was maintained without differences between the two groups. Only the Chair Stand Test (a proxy for muscle strength) declined in the group with CI. Similarly, the proportion of participants with balance impairment did not improve in the CI group at 6-months. In contrast, for the non-CI group, there was a trend toward improvement, although not statistically significant *(*Table 3*)*.

**Table 3:** Differences on physical performance at 3 and 6-months follow-up. Analysis stratified by presence of cognitive impairment (CI).

	3-months follow-up			6-months		
	CI	Non-CI	p-value	CI	Non-CI	p-value
	*Difference from baseline*	*Difference from baseline*		*Difference from baseline*	*Difference from baseline*	
**SPPB; mean (SD)**	1.1 (1.8)	1.6 (1.8)	0.094	0.86 (1.93)	1.31 (2.2)	0.294
**Gait speed (m/s); mean (SD)**	0.05 (0.13)	0.09 (0.13)	0.088	0.04 (0.14)	0.05 (0.16)	0.069
**Chair Stand Test (s); mean (SD)**	-2.56 (11.43)	-6.11 (12.10)	**0.042**	-0.34 (12.06)	-6.75 (2.12)	**0.033**
**Balance impairment, n (%)**	15 (18.3)	26 (23.2)	0.407	7 (8.5)	12 (10.7)	0.614

SD: standard deviation; a. SPPB: Short Physical Performance Battery, range from 0 to 12. T-test for or Wilcoxon signed-rank was used for continuous variables as appropriated and Chi-square for categorical variables. For the “balance impairment and falls categories, we report the n (%) of participants with a positive change

**Fig. 2:**
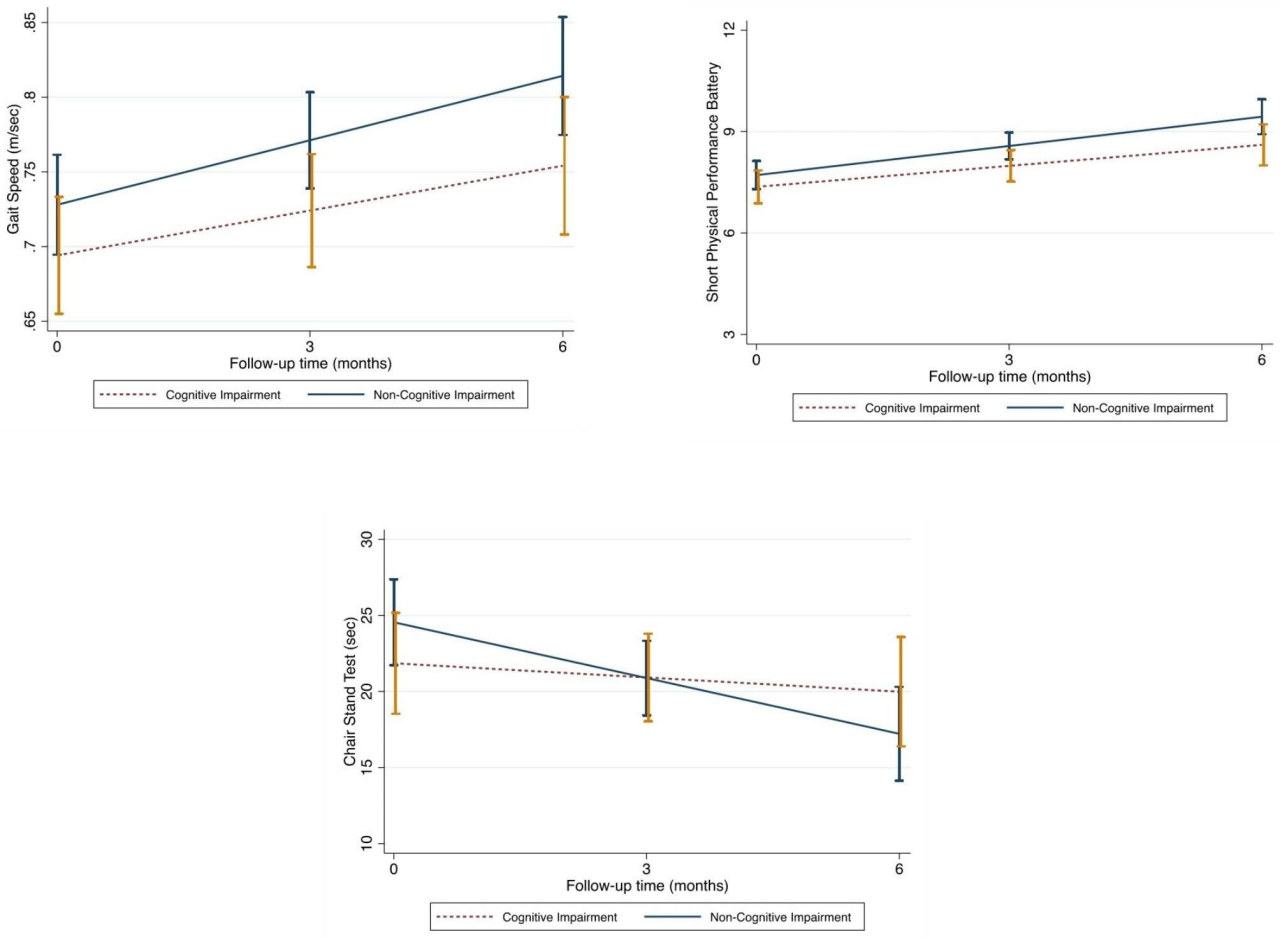
Predicted physical performance variables over the 6-month follow-up in relation to the presence of cognitive impairment or dementia at baseline. ß coefficients for the interaction term between time and the exposure, obtained through linear mixed models. Models adjusted by sex, age, education level, depression, and functional capacity at baseline. p-value for interaction between cognitive impairment x time to predict gait speed performance: 0.217. p-value for interaction between cognitive impairment x time to predict Short Physical Performance Battery results: 0.333. p-value for interaction between cognitive impairment x time to predict chair stand test performance: 0.035

Looking at the impact of CI on the monthly change in physical performance measures across the 6-month follow-up, CI had no statistically significant impact on the improvement of SPPB or gait speed. In contrast, it was associated with a worst performance in the Chair stand test (Fig. 2).

## Discussion

In our community-dwelling older adult population, a tailored multifactorial intervention (i.e., +AGIL Barcelona), which includes a MEP, had a positive impact at 3-months on the physical function, regardless of CI. The overall improvement appeared to be maintained for up to six months.

 [42] The evidence regarding the benefits of multifactorial interventions to reduce or revert frailty is strong. The WHO recently developed a guide for healthcare professionals aiming to perform a comprehensive assessment in primary care settings and, according to the deficits, recommends possible interventions to follow. The + AGIL program offers a multidisciplinary and multi-component, person-centered intervention for older adults with frailty, independently of their cognitive status. Our results, in both participants with and without CI, achieved statistical significance and clinically meaningful improvements in physical function [43]. A systematic review and meta-analyses by Lam F et al. [18] showed solid evidence that supervised exercise training improves physical performance in older adults with CI. In terms of intensity, the authors reported that a minimum of 60 min per session, 2 to 3 days per week, effectively improve various aspects of physical functioning, especially in those with poorer physical function. As a substantial difference, compared to our study, only 44% of participants were recruited from the community, whereas 47% were recruited from nursing homes. This means that the enrolled population had a higher degree of dependency and an increased likelihood of receiving a greater intensity of the intervention.

Regarding the duration of the benefits over time, Cadore et al. [17] reported an initial improvement in muscle strength, balance and gait ability, and incidence of falls after an 8-week MEP in institutionalized older adults with dementia. However, after 24 weeks of training cessation, there was a significant decrease in most of the outcomes assessed, with a substantial decrease in physical condition, at even worse levels compared with the pre-training status. We can speculate that the differences observed in our program could be attributable to our sample’s relatively preserved functional status, compared to the advanced conditions of Cadore’s study. However, the specific aim of the + AGIL program to promote healthy habits using motivational interviewing and the integration of community resources to foster the continuation of PE practice might influence the sustainability of our results.

The cross-sectional relationship between cognitive and physical function has also been widely reported in systematic reviews [44]. Older adults with slow gait speed tend to have worse cognition, and an increased risk of cognitive decline and dementia development [45]. A negative association between CI and physical impairments has also been described. Physical dysfunction and cognitive impairment have been put together to contextualize the construct of Motoric Cognitive Risk Syndrome (MCR) [46], a heterogeneous clinical manifestation characterized by the simultaneous presence of both slow gait and cognitive complaints that can represent a precursor of neurodegenerative processes, and may be potentially reversible.

Not surprisingly, our results with + AGIL intervention showed an improvement in physical function (gait speed, SPPB) in older adults with and without CI, without differences between both groups. Moreover, our participants with some degree of CI, after improving in all the physical performance tests, presented a slight decrease in the Chair-Stand-Test, which has also been described as significantly related to global cognitive performance [42]. Previous studies also describe a protective effect of physical exercise on cognition, improving cerebral perfusion and increasing neurogenesis [47, 48, 49], leading to a possible inverse relationship between exercise and the risk of developing dementia [50, 51, 52, 53]. Additionally, interventions including MEP in hospitalized [54], community-dwelling [19] and institutionalized [17] older adults have shown that physical exercise and physical activity [55] can improve cognition, muscle strength, balance, and gait ability and reduce falls risk, even in older adults with CI or dementia [56]. In consequence, exercise seems to be an effective intervention to revert or minimize the physical consequences of mild cognitive impairment and dementia. Unfortunately, we could not test the impact of exercise on change in cognitive function over time, due to the limited longitudinal follow-up on cognition and the low sensitivity to change of the Mini-Cog©.

We acknowledge some limitations of this study. First, we use the Mini-Cog©, which has high sensitivity as a screening tool, supported by the Gerontological Society of America to improve CI detection in primary care and referral to specialized dementia units. However, diagnosing or follow-up cognitive function in a population with an established CI is not appropriate. So, from one hand, we could not fully characterize CI at baseline (type, severity, potential diagnosis of dementia), nor this tool is sensitive enough to capture changes in cognition in response to exercise. Second, the study did not include a control intervention. Finally, up to 40% of our missing participants were due to the COVID-19 pandemic, where all outpatient clinics were suspended for almost a year, limiting the statistical power of the six months results.

Among strengths, the + AGIL Barcelona is a research-implementation program developed using a co-design approach (including participants and caregivers with cognitive impairment) and implemented in the “real world” population, favoring the program’s high adherence. Second, the holistic approach is based on the CGA. Third, we report longitudinal results of a well-characterized population of dwelling older adults.

## Conclusions

Our study suggests that a multicomponent intervention, the + AGIL Barcelona program, can be delivered in older adults with cognitive impairment and be as effective as it is for cognitively intact older adults in terms of physical function (SPPB and gait speed). Although further studies with an experimental design are warranted, our results reinforce the need to implement MEP, embedded in holistic interventions based on the CGA, in community-dwelling older adults with CI. Moreover, support the need to include physical performance and functional assessment as part of the CGA in daily clinical practice, especially in older persons with CI.

## Data Availability

The datasets generated and/or analyzed during the current study are not publicly available due to the sensitive nature of the personal health data collected from a vulnerable population and privacy and confidentiality reasons, but might be partially available from the corresponding author on reasonable request.

## List of abbreviations

CI: Cognitive impairment
PE: Physical exercise
PA: Physical activity
RTC: Randomized control trial
CGA: Comprehensive Geriatric Assessment
GFST: Gerontopôle Frailty Screening Tool
MEP: Multicomponent exercise program
SPPB: Short Physical Performance Battery
GS: Gait speed
SD: Standard Deviation
IQR: Interquartile range
MCR: Motoric Cognitive Risk Syndrome
